# Implementation of a Metered-Dose Inhaler to Dry-Powder Inhaler National Formulary Transition

**DOI:** 10.1001/jamanetworkopen.2024.49234

**Published:** 2024-12-05

**Authors:** Alexander S. Rabin, Julien B. Weinstein, Taylor N. Whittington, Cainnear K. Hogan, Deborah Khachikian, Sarah M. Seelye, Hallie C. Prescott

**Affiliations:** 1 Pulmonary Section, Veterans Affairs Ann Arbor Healthcare System, Ann Arbor, Michigan; 2 Department of Internal Medicine, University of Michigan, Ann Arbor; 3 Veterans Affairs Center for Clinical Management Research, Ann Arbor, Michigan; 4 Veterans Affairs Pharmacy Benefits Management Services, Hines, Illinois

## Abstract

This quality improvement study analyzes prescribing patterns among US Department of Veterans Affairs facilities after a July 2021 metered-dose inhaler (budesonide-formoterol) to dry-powder inhaler (fluticasone-salmeterol) national formulary transition.

## Introduction

Metered-dose inhalers (MDIs) containing hydrofluorocarbon propellants have a greater carbon footprint than dry-powder inhalers (DPIs),^1^ which do not require an aerosol propellant for drug delivery. Despite calls to reduce inhaler-related greenhouse gas emissions,^2^ data on the large-scale implementation of device switching from MDIs to DPIs are limited.

In July 2021, following a competitively bid contract negotiation, the US Department of Veterans Affairs (VA) transitioned from the budesonide-formoterol MDI (Symbicort; AstraZeneca) to the fluticasone-salmeterol DPI (Wixela Inhub; Viatris) as its preferred combination inhaled corticosteroid and long-acting β_2_-agonist controller medication.^3^ We analyzed trends in inhaler prescribing patterns among VA facilities following the national formulary transition.

## Methods

Data on inhaler dispensing from January 1, 2018, to December 31, 2022, were extracted from the VA Corporate Data Warehouse. The study was reviewed by the VA Ann Arbor Institutional Review Board and deemed exempt from the need for informed consent according to 45 CFR §46, category 4. All controller inhalers in the VA national formulary antiasthma therapeutic class (RE109)^4^ containing 2 or more medication classes (inhaled corticosteroid, long-acting β_2_-agonist, or long-acting muscarinic antagonist) were included (hereinafter, combination inhalers). We determined overall and facility-specific rates of switching to the fluticasone-salmeterol DPI among patients prescribed a combination inhaler before and after the formulary transition in July 2021, and of subsequent switching away from the fluticasone-salmeterol DPI. Nonformulary drug consultation requests for combination inhalers after the formulary transition, the outcome of the nonformulary consultation (ie, approval or denial), and the reason for the determination were examined. Analyses were performed with Stata, version 18.0 (StataCorp). The study followed the SQUIRE reporting guideline.

## Results

Of 347 486 patients (mean [SD] age, 65.8 [11.9] years; 319 230 men [91.9%] and 28 256 women [8.1%]) who were dispensed a combination inhaler before and after the formulary transition, 260 268 (74.9%) were switched to the fluticasone-salmeterol DPI. Of these switched patients, 37 036 (14.2%) were subsequently prescribed a non–fluticasone-salmeterol combination inhaler. The Figure illustrates proportions of patients switched to the fluticasone-salmeterol DPI (by VA facility) and switched back to a non–fluticasone-salmeterol combination inhaler.

**Figure.**
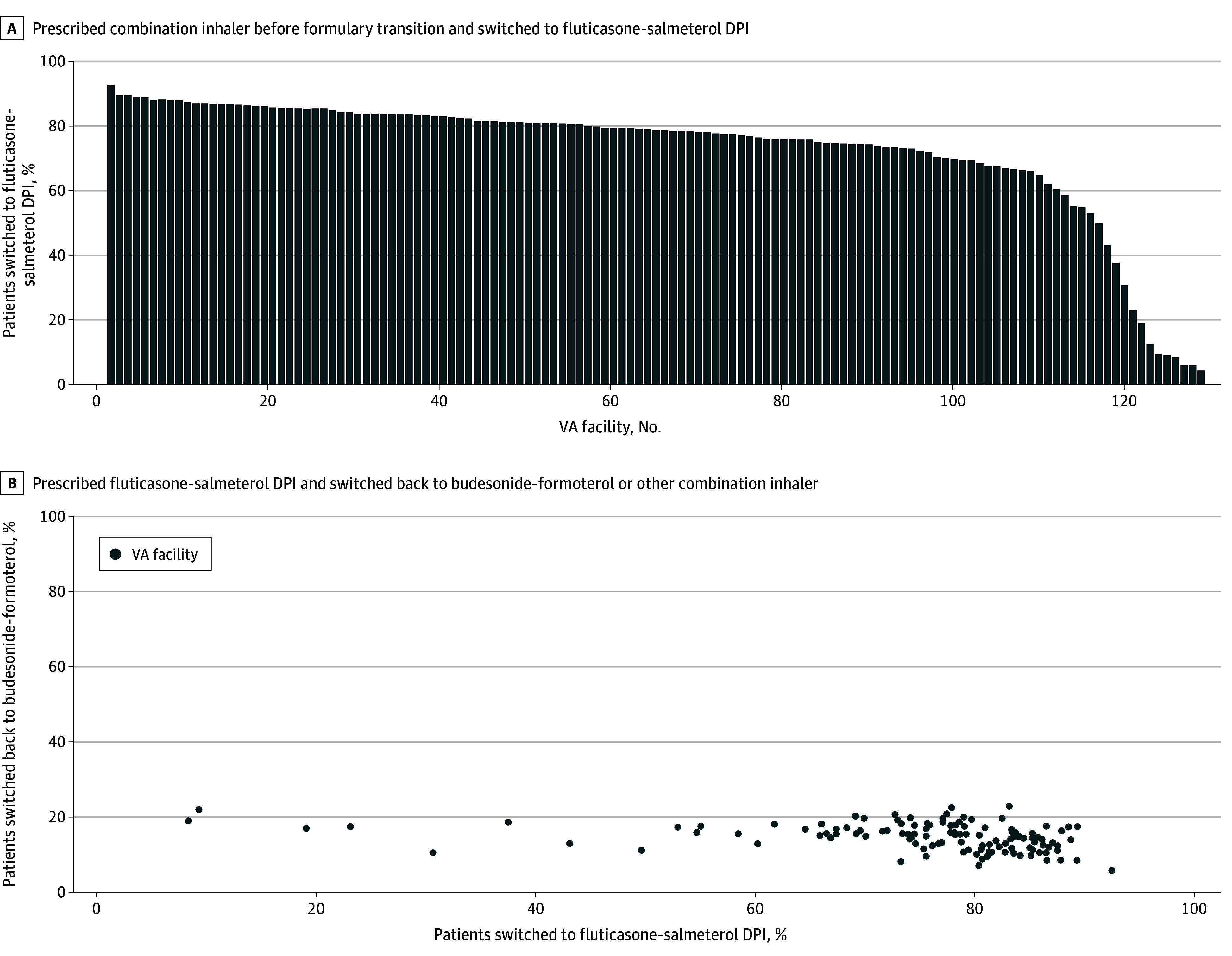
Patients Switched to a Fluticasone-Salmeterol Dry-Powder Inhaler (DPI) by US Department of Veterans Affairs (VA) Facility and Patients Switched Back to Budesonide-Formoterol or Another Combination Inhaler Following the VA National Formulary Transition Although the VA formulary is determined centrally, it is implemented locally at the facility level. A, Proportion of patients prescribed a combination inhaler prior to the formulary transition who were switched to the fluticasone-salmeterol DPI (Wixela Inhub; Viatris) following the VA national formulary transition in July 2021. Each bar represents 1 VA facility. B, Proportion of patients prescribed the fluticasone-salmeterol DPI who were switched back to budesonide-formoterol (Symbicort; AstraZeneca) or another combination inhaler. Each dot represents 1 VA facility. Most VA facilities switched more than 80% of patients receiving combination inhalers to the fluticasone-salmeterol DPI, but a small proportion of facilities had much lower switch rates. Regardless of switch rate, however, 14.2% of patients were switched back to an alternate inhaler.

Among 23 350 VA nonformulary consultations, there were 10 481 requests for budesonide-formoterol and 12 869 requests for other combination inhalers; of these, 8713 (83.1%) and 12 628 (98.1%) were approved, respectively (Table). The most common reason for nonformulary consultation approval was a therapeutic failure of the fluticasone-salmeterol DPI, and the most common reason for disapproval was that a trial of a preferred alternative device had not been exhausted.

**Table.** Nonformulary Consultation Requests for Budesonide-Formoterol and Other Combination Inhalers Following the VA National Formulary Transition From a Budesonide-Formoterol MDI to a Fluticasone-Salmeterol DPI, 2021-2022^a^

Reason for approval or disapproval	No. (%) of nonformulary consultation requests		
	For budesonide-formoterol (n = 10 481)	For other combination inhalers (n = 12 869)^b^	For budesonide-formoterol and other combination inhalers (n = 23 350)^b^
Request approved^c^	8713 (83.1)	12 628 (98.1)	21 341 (91.4)
Therapeutic failure of formulary medication	6236 (71.6)	9428 (74.7)	15 664 (73.4)
Adverse reaction to formulary medication	2232 (25.6)	2972 (23.5)	5204 (24.4)
Symptoms previously responded to selected medication and serious risk with change to formulary medication	606 (7.0)	126 (1.0)	732 (3.4)
No formulary alternative exists	384 (4.4)	708 (5.6)	1092 (5.1)
Meets VA continuity of care criteria	287 (3.3)	239 (1.9)	526 (2.5)
Contraindication to formulary medication	159 (1.8)	196 (1.6)	355 (1.7)
Request disapproved	1767 (16.9)	238 (1.9)	2005 (8.6)
Preferred alternatives were not exhausted	1480 (83.8)	146 (61.3)	1626 (81.1)
Maximum dose of preferred medication not trialed	225 (12.7)	39 (16.4)	264 (13.2)
Indication not supported	183 (10.4)	65 (27.3)	248 (12.4)
Nonadherence to preferred medication	49 (2.8)	15 (6.3)	64 (3.2)
Contraindication to requested medication	1 (0.1)	3 (1.3)	4 (0.2)

Abbreviations: DPI, dry-powder inhaler; HFA, hydrofluoroalkane; MDI, metered-dose inhaler; VA, US Department of Veterans Affairs.

^a^ The VA national formulary transition from the budesonide-formoterol MDI (Symbicort; AstraZeneca) to the fluticasone-salmeterol DPI (Wixela Inhub; Viatris) occurred in July 2021. Data are presented for July 1, 2021, to December 31, 2022.

^b^ Combination inhalers included mometasone-formoterol (Dulera HFA; Organon) (n = 10 173), budesonide-glycopyrrolate-formoterol (Breztri Aerosphere; AstraZeneca) (n = 2385), fluticasone-vilanterol (Breo Ellipta; GlaxoSmithKline) and umeclidinium-vilanterol (Anoro Ellipta; GlaxoSmithKline) (n = 147), fluticasone-salmeterol (Advair Diskus; GlaxoSmithKline; and AirDuo RespiClick; Teva) (n = 138), fluticasone-salmeterol (Advair HFA; GlaxoSmithKline) (n = 16), fluticasone-umeclidinium-vilanterol (Trelegy Ellipta; GlaxoSmithKline) (n = 8), and tiotropium-olodaterol (Stiolto Respimat; Boehringer Ingelheim) (n = 2).

^c^ Four nonformulary consultations were neither approved nor disapproved. Percentages do not sum to 100% because more than 1 reason could be given for approval or disapproval.

## Discussion

A VA national formulary transition in 2021, favoring a DPI (fluticasone-salmeterol) over an MDI (budesonide-formoterol), resulted in marked changes in inhaler prescribing across the VA health care system, with swift uptake of the DPI among a majority of facilities. Following implementation of this formulary change, fewer than 1 in 6 patients (37 036 of 260 268 [14.2%]) were switched back to budesonide-formoterol or another nonformulary combination inhaler, and the rate of switchback was consistent across VA facilities.

This study has some limitations. Although most veterans who have filled a medication through the VA rely on the VA alone for prescription medications,^5^ we did not explore prescribing outside of the health care system and therefore may have underestimated inhaler device switching after the formulary transition.

As health care systems and payers consider substituting MDIs with DPIs to mitigate the climate impact of respiratory care,^2^ our results suggest that, in a large sample of veterans with a high burden of respiratory disease,^6^ a device transition may be carried out with a relatively low failure rate (ie, requiring a transition back to an MDI). Additional research is needed to understand the effects of inhaler device switching on respiratory health outcomes, health care use, and net health care–related greenhouse gas emissions, as well as optimal methods to support a successful device transition.

## Notes

Supplement.
Data Sharing Statement
