# Supplementary material for: Implementation of a Metered-Dose Inhaler to Dry-Powder Inhaler National Formulary Transition
Source: JAMA Netw Open. 2024 Dec 5;7(12):e2449234. doi: 10.1001/jamanetworkopen.2024.49234 (PMC11621982; doi:10.1001/jamanetworkopen.2024.49234)
Supplement: Supplement. — Data Sharing Statement [file jamanetwopen-e2449234-s001.pdf]

Data Sharing Statement

Rabin. Implementation of a Metered-Dose Inhaler to Dry-Powder Inhaler National Formulary Transition. *JAMA Netw Open*. Published December 05, 2024.
doi:10.1001/jamanetworkopen.2024.49234

Data
Data available: No
